# Primary renal leukaemia in a young adult male as an extramedullary presentation of T cell acute lymphoblastic leukaemia

**DOI:** 10.1002/jha2.820

**Published:** 2023-11-15

**Authors:** Punit Jain, Poonam Jain, Robert S. Ohgami, Veena Pawar, Kunal Sehgal, Pradnya Chaudhari, Ravindra Nikalji, Tejinder Singh, Vipin Khandelwal, Sanjay Khare, Vaishali Lokhande, Ashwathy Haridas, Laxman Jessani, Kanika Khandelwal

**Affiliations:** ^1^ Haematology Oncology and Stem Cell Transplant Unit Apollo Hospitals Navi Mumbai India; ^2^ Helix Genetic and Pathology Laboratory Mumbai India; ^3^ Hematopathology University of Utah Salt Lake City Utah USA; ^4^ Hematopathology Apollo Hospitals Navi Mumbai India; ^5^ Hematopathology Sehgal Path Lab Private Limited Mumbai India; ^6^ Nephrology Apollo Hospitals Navi Mumbai India; ^7^ General Medicine Apollo Hospitals Navi Mumbai India; ^8^ Infectious Disease Apollo Hospitals Navi Mumbai India

**Keywords:** acute renal failure in T‐ALL, extramedullary manifestations in T‐ALL, Modified BFM‐90 protocol, T cell acute lymphoblastic leukaemia

## Abstract

Primary renal involvement by T lymphoblasts is rare among adults with T acute lymphoblastic leukaemia. We report a 28‐year‐old man presenting with acute renal failure due to infiltration by T lymphoblasts and his response to paediatric‐inspired modified BFM‐90 protocol. The patient achieved an initial complete remission (CR) but developed central nervous system relapse. He achieved CR2 with cranial irradiation and intrathecal chemotherapy. He underwent a haploidentical transplant in CR2 and remains in remission post‐transplant day 330. An early kidney biopsy helped confirm the diagnosis. Such presentations remain responsive to modified BFM‐90. An early allotransplant in CR2 remains the standard of care.

## 1 BACKGROUND

Extramedullary (EM) presentations of acute leukaemia include lymphadenopathy, central nervous system disease, mediastinal mass, testicular swelling, and other organ infiltrations [1, 2]. Although EM presentations have been reported commonly in acute myeloid leukaemias (AMLs) with an incidence rate of up to 23.7%, they lack independent prognostic significance in AML [2]. Primary renal involvement by the leukaemic blasts has been reported among children with T‐cell acute lymphoblastic leukaemia (T‐ALL) in up to 19.5% [1]. However, its incidence in adults with T‐ALL is unknown [1]. Primary renal presentations in children with ALL include acute renal failure, hematuria, nephromegaly or hemolytic uremic syndromes [1]. Isolated primary renal failure involvement among adults in T‐ALL and its role as a prognostic factor, when treated with the modified BFM90 protocol, has rarely been reported [3, 4]. Here, we describe the demographics and clinical course of a rare primary renal presentation of an acute T cell lymphoblastic leukaemia in a young man from India and its one‐year outcome following a T‐replete haploidentical stem cell transplant.

## 2 CASE PRESENTATION

A 28‐year‐old man was referred to Apollo Hospitals, Navi Mumbai, in November 2021, with a history of intermittent fever, vomiting, and bilateral painless swelling in the neck for one week. The initial workup showed cytopenia [Haemoglobin 12.1 g %, White blood count 2.22 × 10^3^/mm^3^ (neutrophils 58%, lymphocytes 27%, monocytes 11%, eosinophils 4%), Platelet count 18 × 10^3^/mm^3^] with features of non‐oliguric acute renal failure (ARF) [blood urea nitrogen 182 mg/dL, creatinine 12.8 mg/dL, potassium 7.7 mmol/dL, lactate dehydrogenase 627 units/L]. Ultrasonography (USG) of the abdomen showed bilaterally enlarged kidneys with increased echogenicity in the bilateral renal medulla.

## 3 DIAGNOSIS

**FIGURE 1:**
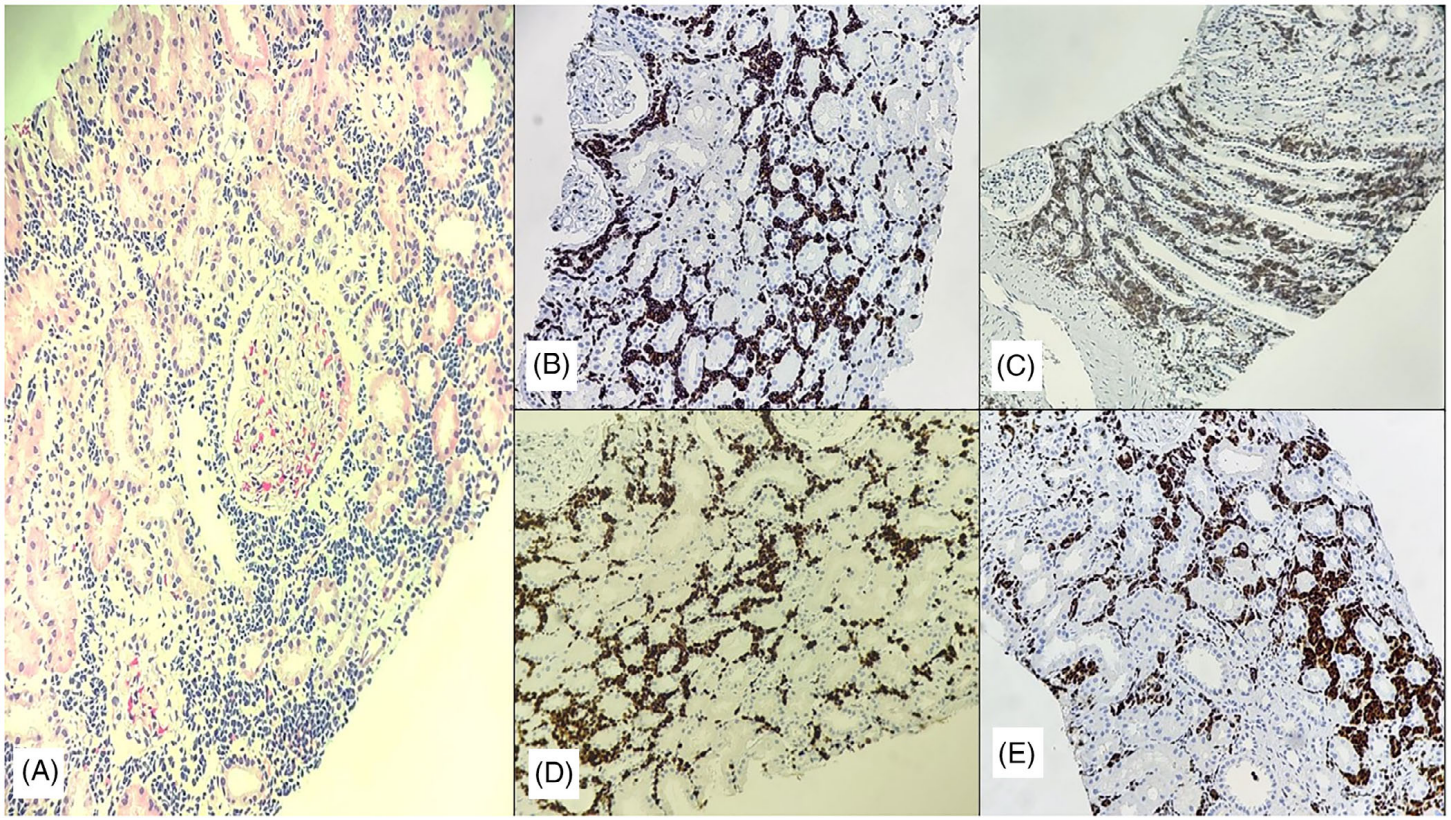
(A) Renal core with unremarkable glomeruli with diffuse infiltration of medium‐sized monotonous atypical lymphoid cells, H & E stain,40X. (B) CD3‐ Positive on lymphoblastic cells, 40X. (C) CD5‐ Positive on atypical cells, 40x. (D) Ki67 â€“ 80%, 40x. (E) TdT‐ Positive on neoplastic cells, > 75% positive.

**FIGURE 2:**
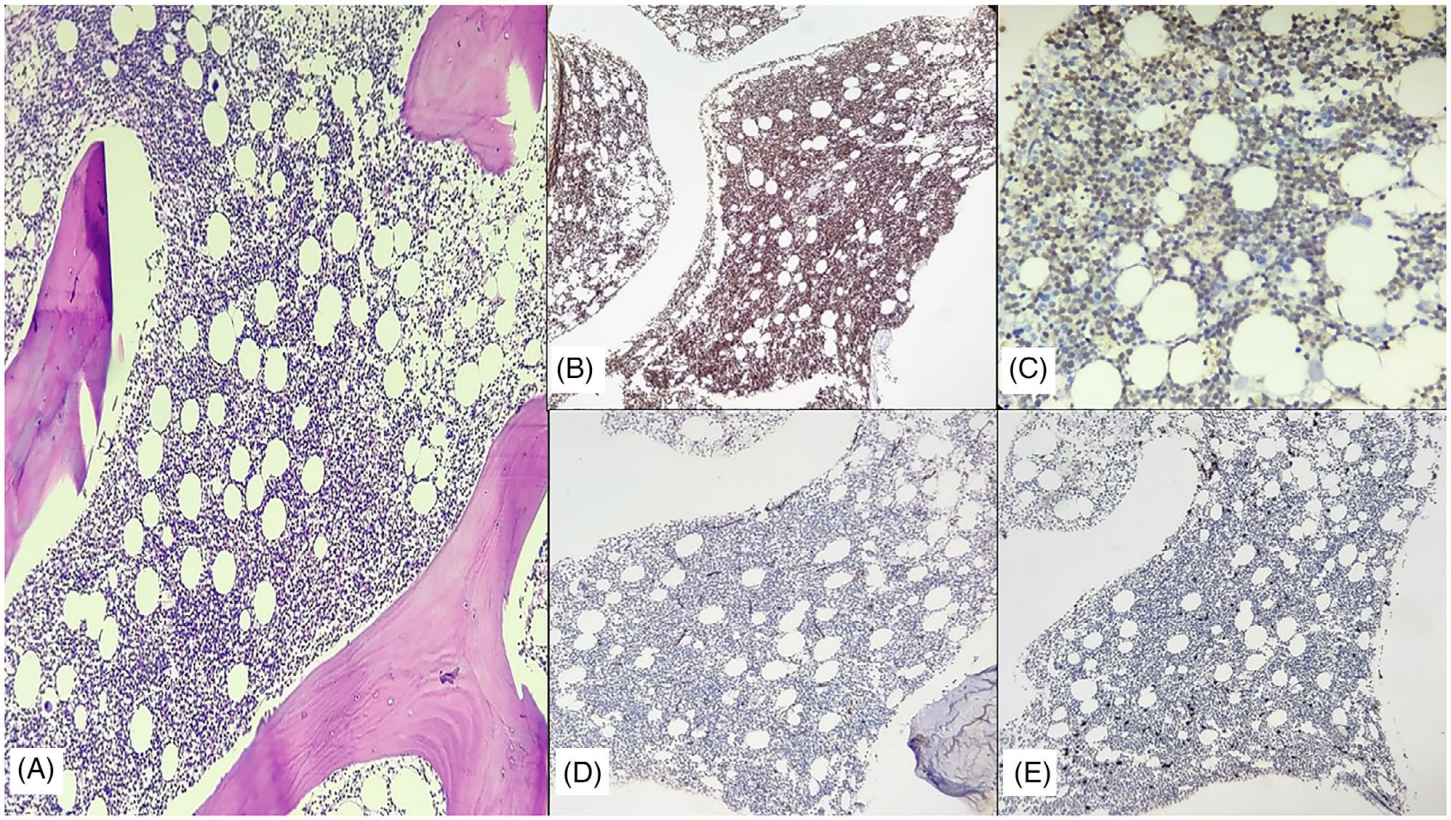
(A) Bone marrow biopsy, cellular marrow with diffuse infiltration by atypical small lymphoid cells, H & E stain,40X. (B) CD3‐ Positive on lymphoblastic cells, 40X. (C) TdT‐ Positive on neoplastic cells, 40X. (D) CD20‐ Negative on lymphoblastic cells, 40X. (E) CD34‐ Negative on lymphoblastic cells, 40X.

The initial bone marrow (BM) aspiration and biopsy with immunohistochemistry at diagnosis showed a hypocellular marrow with decreased erythropoiesis and myelopoiesis without blasts. (Figure S1). The flow cytometry (FCM) did not show any abnormal blasts. Given the above findings, a kidney biopsy was done under adequate platelet cover, which confirmed its T‐cell lymphoblastic infiltration (Figure 1A–E). Due to persistent cytopenia, a repeat BM done 11 days after the first BM showed 55% lymphoblasts, and FCM confirmed a T‐ALL (CD7+, CD2+, CD3+, CD4+, CD8+, CD5+ and CD38+) (Figure 2A–E). The cerebrospinal fluid (CSF) assessment was normal. (Fluid count and gross examination—Colourless fluid with only one lymphocyte seen; Cytospin for malignant cells was negative). The conventional karyotype was normal, and no high‐risk (HR) markers were detected on fluorescent in situ hybridisation (FISH). The next‐generation sequencing using BM samples showed the presence of NOTCH (Variable allele frequency 33.5%) mutations. Other mutations of uncertain significance included SBDS, KMT2D and PARN mutations.

## 4 TREATMENT

He was initially stabilised with haemodialysis for his severe hyperkalaemia and renal dysfunction. He was started on modified adult BFM‐90 protocol in November 2021, with a complete remission (CR) [minimal residual disease (MRD) negative by FCM] status post‐induction and total resolution of ARF. A repeat USG/colour doppler of the abdomen at the end of induction showed bilaterally normal kidney position and size. They showed normal cortical reflectivity and corticomedullary distinction, with no other evidence of hydronephrosis or mass effect.

Post phase II induction, he was initiated on consolidation with high‐dose methotrexate consolidation (3 g/m^2^). After three cycles of consolidation, he developed a prolonged cytopenia lasting almost 3 weeks. BM on‐count recovery remained in CR, but CSF analysis confirmed an isolated EM relapse in the central nervous system (Fluid count and gross examination—Colourless fluid with 477 cells/μL (neutrophils – 9%, lymphocytes – 27%, atypical cells – 64%, Cytospin for malignant cells was positive). He received cranial radiation (RT) (24 gray) with triple intrathecal chemotherapy (TIT) (injection methotrexate 12 mg, injection hydrocortisone 50 mg and injection cytarabine 50 mg). He was then started on HyperCVAD (Arm B—Methotrexate arm = 1 g/m^2^), again followed by a prolonged cytopenia. On haematological recovery, he remained in CR. He did have two episodes of severe life‐threatening neutropenic sepsis. Considering the HR nature of his disease and his current MRD‐negative CR2 status, he underwent a T‐replete haplotransplant (mother being the donor) in October 2022 with injection fludarabine (150 mg/m^2^), injection treosulfhan (36 g/m^2^), and total body irradiation (2 gray) [5]. Stem cell dose infused (CD34) was 6.8 × 10^6^ cells/kilograms (kg). Graft versus host (GVH) prophylaxis included post‐transplant cyclophosphamide (50 mg/kg/day on day +3, day+4), oral mycophenolate mofetil (15 mg/kg thrice daily from day +5 to day +30) and oral tacrolimus (0.03 mg/kg/day in two divided dosed from day +5 onwards). His peri transplant period was uncomplicated, with only minor infections and minimal mucositis grade I. He achieved 100% donor chimerism on day 28 by FISH and short tandem repeat analysis. He did develop grade III acute gut GVHD and later chronic skin GVHD requiring prolonged steroids and oral ruxolitinib. He also developed severe cytomegalovirus (CMV) viremia requiring ganciclovir therapy and BK virus hemorrhagic cystitis requiring cidofovir three months post‐transplant, treated as per established guidelines [6, 7].

## 5 OUTCOME AND FOLLOW‐UP

He is currently day + 330 post‐transplant with a stable chronic skin GVHD, on tapering low‐dose prednisolone and ruxolitinib. Blood CMV titres are undetectable, and he remains in CR with a good quality of life despite a very early relapse while on consolidation with high‐dose methotrexate therapy.

## 6 DISCUSSION

Renal involvement as a primary manifestation in adult T‐ALL is rare, and its long‐term impact on the residual renal function and the prognosis of the disease is uncertain. In contrast, such renal involvement in children has not been considered an unfavourable prognostic maker [1]. We believe using pediatric‐inspired regimens like the modified BFM‐90 can result in similar long‐term outcomes in younger adults and children [8].

The current T‐ALL risk stratification in adults is more genomic, with a limited role for complex karyotypic abnormalities (≥ three cytogenetic alterations) in a few studies [9, 10]. Although NOTCH mutations show a favourable prognostic impact in children treated with ALL BFM regimens, their positive impact among adults is limited to the GRALL 03/05 study. Another study on T‐ALL by UKALLXII/E2993 and GMALL 05/93 and 06/99 did not show a favourable outcome with NOTCH mutations. In our patient, we used flow‐based MRD assessment for post‐induction response assessment in T‐ALL [11]. Thus, despite its EM presentation, our patient was initially stratified as standard risk ALL based on a negative end induction MRD and the absence of unfavourable genetics (Absence of NOTCH mutations, presence of RAS and/or PTEN mutations) [12].

The long‐term overall survival rate in adults with T‐ALL is between 48% and 60.9% in CR1, but patients with a relapse or refractory T‐ALL have a median survival of less than eight months despite the best efforts [3, 4, 8]. Relapsed T‐ALL remains a complex disease and lags in translational medicine compared to patients with relapsed B‐ALL; hence, its outcome remains poor [13]. Although nelarabine has been approved for relapsed T‐ALL in adults, the lack of easy access and financial restrictions in developing countries like India limits its use. Our patient responded well to the initial modified BFM90 chemotherapy. Still, he had an EM relapse involving the central nervous system (CNS) during consolidation. Isolated CNS relapse in patients presenting with T‐ALL and renal manifestations at primary diagnosis has been reported in only two prior studies involving children [1].

He achieved CNS remission with cranial RT + TIT, later followed by salvage therapy with HyperCVAD (arm B One cycle only). Since he was in CR2 (MRD negative) with just one cycle, he underwent a haplotransplant. Allogeneic transplants in early CR2 have demonstrated longer disease‐free survivals (D.F.S) in such patients [14]. He has maintained remission for the past 11 months post‐transplant.

Our patient also developed significant gut and skin GVHD, which could be attributed to the female donor effect, the use of peripheral blood graft and the CR2 status followed by a myeloablative regimen [15]. More evidence is needed to determine whether the graft versus leukaemia (GVL) effect also assisted in maintaining long‐term remissions in ALL.

## 7 CONCLUSION

Renal presentation of leukaemia is uncommon and offers a significant diagnostic dilemma for the treating clinician. An early kidney biopsy remains an essential step in the diagnosis of an unexplained renal failure. Pediatric‐inspired regimens like modified BFM‐90 can induce desired remission even in such EM presentations of leukaemia. Although complications like relapse, life‐threatening sepsis, GVHD and financial limitations offer additional challenges, our case can still be a model case for managing such HR leukaemias in the real world. Achievement of MRD negativity remains the primary goal for the initial therapy, and an early allotransplant in CR2 remains vital for longer disease‐free survival after relapse.

At the last follow‐up on 10 September 2023, he remained in CR, has a healthy renal function, and has a reasonable quality of life.

## AUTHOR CONTRIBUTIONS

Punit Jain performed the research and its design and wrote the manuscript. Poonam Jain designed the research study and wrote the manuscript. Robert Ohgami contributed essential images. Veena Pawar contributed essential images. Kunal Sehgal provided the concept and editing of the manuscript. Pradnya Chaudhri contributed to the editing of the manuscript. Ravindra Nikhalji contributed to the editing of the manuscript. Tejinder Singh contributed to the editing of the manuscript. Vipin Khandelwal contributed to the editing of the manuscript. Sanjay Khare contributed to the editing of the manuscript. Vaishali Lokhande contributed to the editing of the manuscript. Ashwathy Haridas contributed to the editing of the manuscript. Jessani Laxman contributed to the editing of the manuscript. Kanika Khandelwal contributed to the editing of the manuscript.

## CONFLICT OF INTEREST STATEMENT

The authors declare no conflict of interest.

## FUNDING INFORMATION

The authors have not received any specific grant for this research from any agency of financing in the public, commercial or not‐for‐profit sectors.

## ETHICS STATEMENT

Informed written consent was obtained from the patient's parent/guardian on their behalf and verbally verified by the patient.

## PATIENT CONSENT STATEMENT

The patient has formally approved using the above clinical material for publication in the ejHaem journal.

## CLINICAL TRIAL REGISTRATION

The authors have confirmed clinical trial registration is not needed for this submission.

## Supporting information

FIGURE S1 Bone marrow biopsy, cellular marrow with trilineage hematopoiesis, no evidence of atypical lymphoid infiltrate, H & E stain, 40X.

## Data Availability

All relevant data for this study included in the manuscript or submitted as supplementary data has been anonymised and is available on reasonable request to the corresponding author.
