# Supplementary figures and images for: Primary renal leukaemia in a young adult male as an extramedullary presentation of T cell acute lymphoblastic leukaemia
Source: EJHaem. 2023 Nov 15;5(1):251–5. doi: 10.1002/jha2.820 (PMC10887257; doi:10.1002/jha2.820)

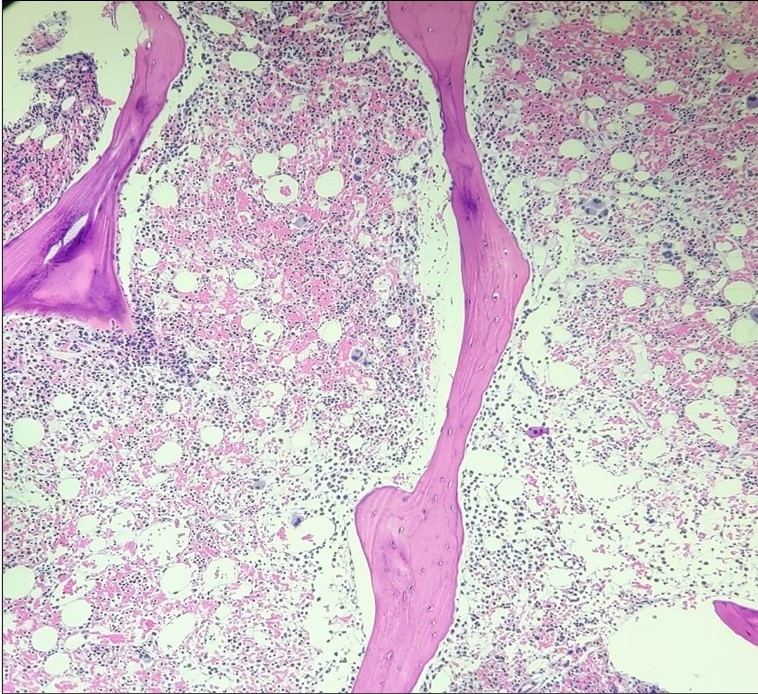

Supplement: Supplementary file 1 — FIGURE S1 Bone marrow biopsy, cellular marrow with trilineage hematopoiesis, no evidence of atypical lymphoid infiltrate, H & E stain, 40X. [file JHA2-5-251-s001.tiff]
